# Supplementary material for: Antimicrobial Activity of Water-Soluble Silver Complexes Bearing C-Scorpionate Ligands
Source: Antibiotics (Basel). 2024 Jul 13;13(7):647. doi: 10.3390/antibiotics13070647 (PMC11273918; doi:10.3390/antibiotics13070647)
Supplement: Supplementary file 1 [file antibiotics-13-00647-s001.zip › antibiotics-2991118-supplementary.pdf]

Antimicrobial activity of water-soluble silver complexes bearing C-scorpionate ligands

Abdallah G. Mahmoud,[1,2] Silvia A. Sousa,[3] M. Fátima C. Guedes da Silva,[1,4]* Luísa M. D. R. S. Martins,[1,4]* Jorge H. Leitão[3]*

[1] Centro de Química Estrutural, Institute of Molecular Sciences, Instituto Superior Técnico, Universidade de Lisboa, Av. Rovisco Pais, 1049–001 Lisboa, Portugal.

[2] Department of Chemistry, Faculty of Science, Helwan University, Ain Helwan, Cairo 11795, Egypt.

[3] Department of Bioengineering (DBE), Institute for Bioengineering and Biosciences (iBB), The Associate Laboratory Institute for Health and Bioeconomy (i4HB), Instituto Superior Técnico (IST), Av. Rovisco Pais, 1049–001 Lisbon, Portugal.

[4] Departamento de Engenharia Química, Instituto Superior Técnico, Universidade de Lisboa, Av. Rovisco Pais, 1049-001 Lisboa, Portugal.

Table S1. Crystallographic data and structure refinement details for **1**.

Empirical formula	$C_{11}H_{14}AgN_7O_5$
Formula Weight	432.16
Crystal system	monoclinic
Space group	C c
Temperature/K	298(2)
a/Å	12.1647(13)
b/Å	19.440(2)
c/Å	8.7652(10)
α/°	90
β/°	130.527(3)
γ/°	90
V (Å^3)	1575.5(3)
Z	4
D_{calc} (g cm^{-3})	1.822
F000	864
μ(Mo Kα) (mm^{-1})	1.319
Rfls. collected/unique/observed	12657 / 2718 / 2495
R_{int}	0.0499
Final $R1$ [a], $wR2$ [b] ($I \geq 2\sigma$)	0.0245, 0.0512
Goodness-of-fit on F^2	1.083

[a] $R = \Sigma||F_o|–|F_c||/\Sigma|F_o|$; [b] $wR(F^2) = [\Sigma w(|F_o|^2 – |F_c|^2)^2/\Sigma w|F_o|^4]^{½}$.

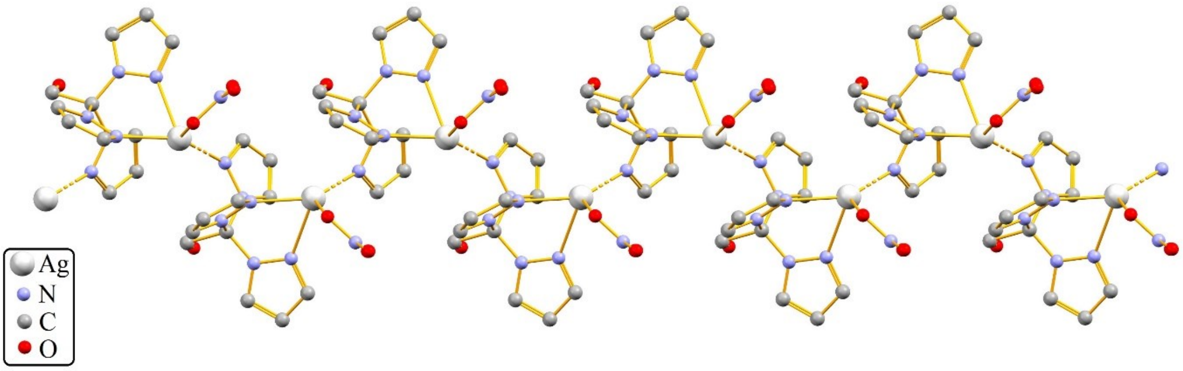

Figure S1. Structural fragment representing the 1D polymer **1**.

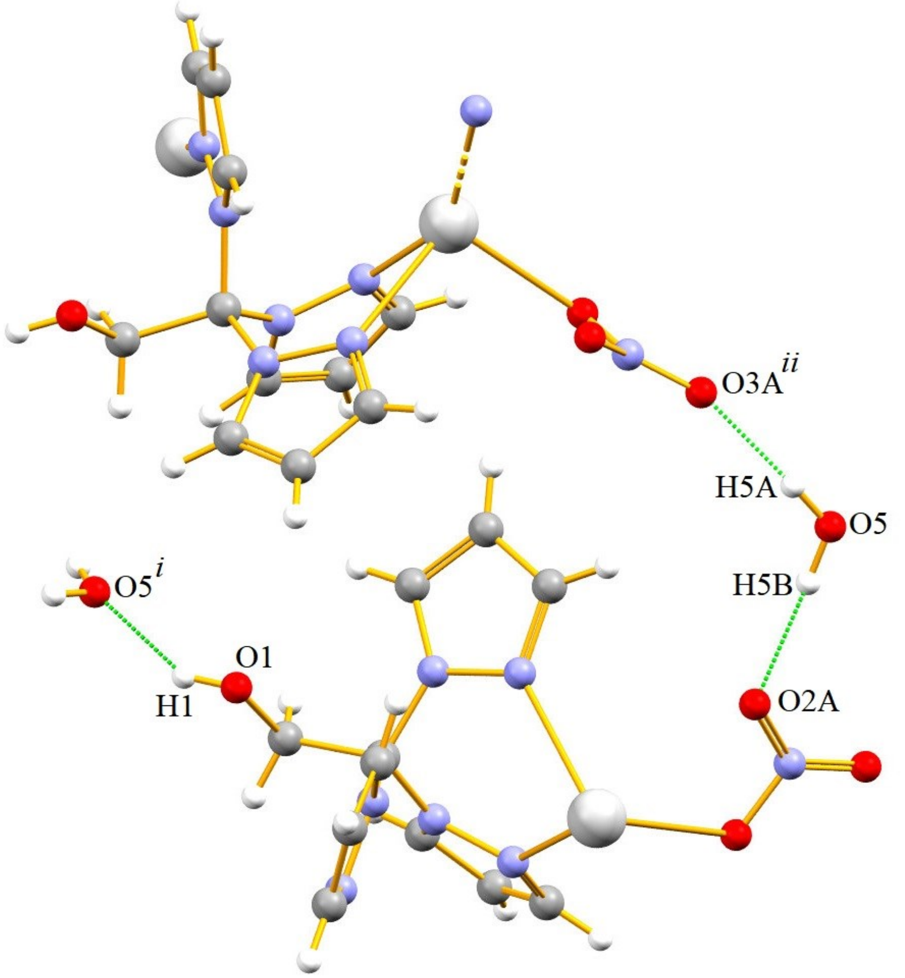

Figure S2. Hydrogen bond interactions ($D_1^1(2)$ graph sets) in the structure of **1** (distances in Å). Symmetry operations to generate equivalent atoms: *i*) -1+x, -y, -1.5+z; *ii*) x,-y,-1/2+z.

D–H···A	D–H	H···A	D···A	D–H···A
O1–H1···O5^{i}	0.820	1.898	2.645(6)	151.1
O5–H5A···O3A^{ii}	0.9(1)	1.88(9)	2.69(3)	150(9)
O5–H5B···O2A	0.9(1)	2.03(9)	2.81(4)	144(10)

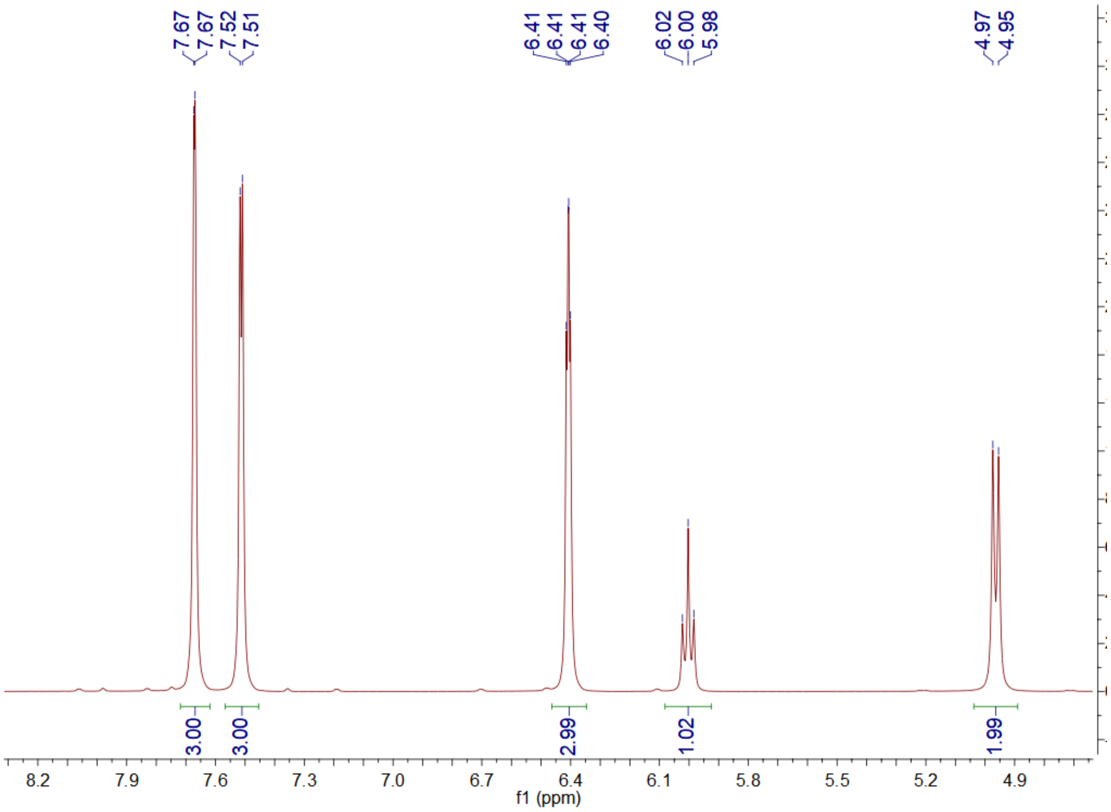

Figure S3. ^{1}H NMR spectrum of complex **1** in DMSO-d_6 (300 MHz).

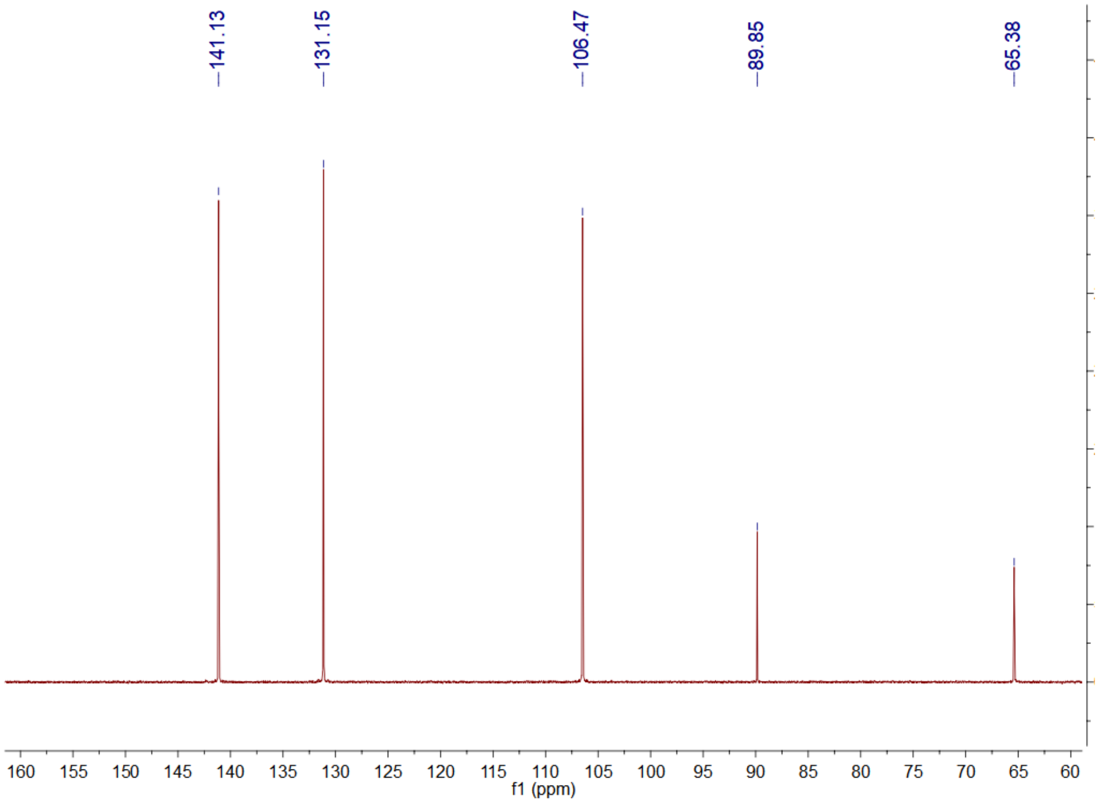

Figure S4. ^{13}C NMR spectrum of complex **1** in DMSO-d_6 (300 MHz).

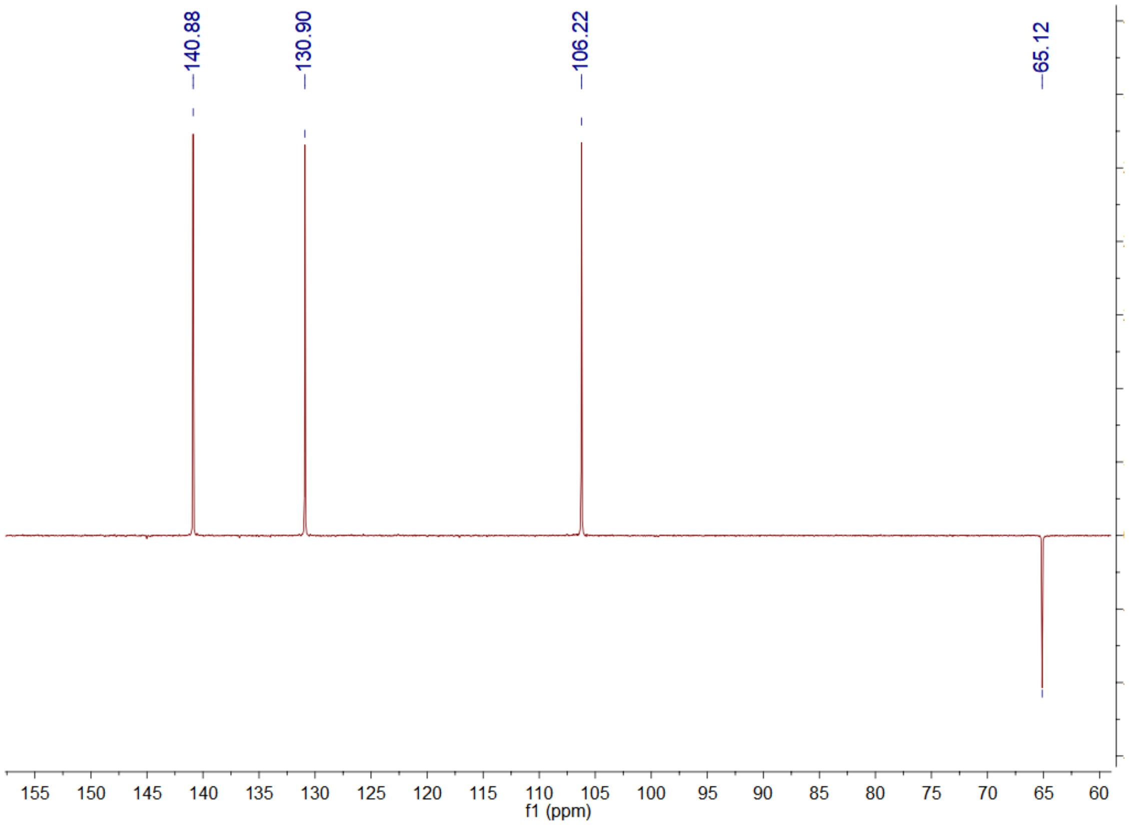

Figure S5. DEPT NMR spectrum of complex **1** in DMSO-*d*$_6$ (300 MHz).

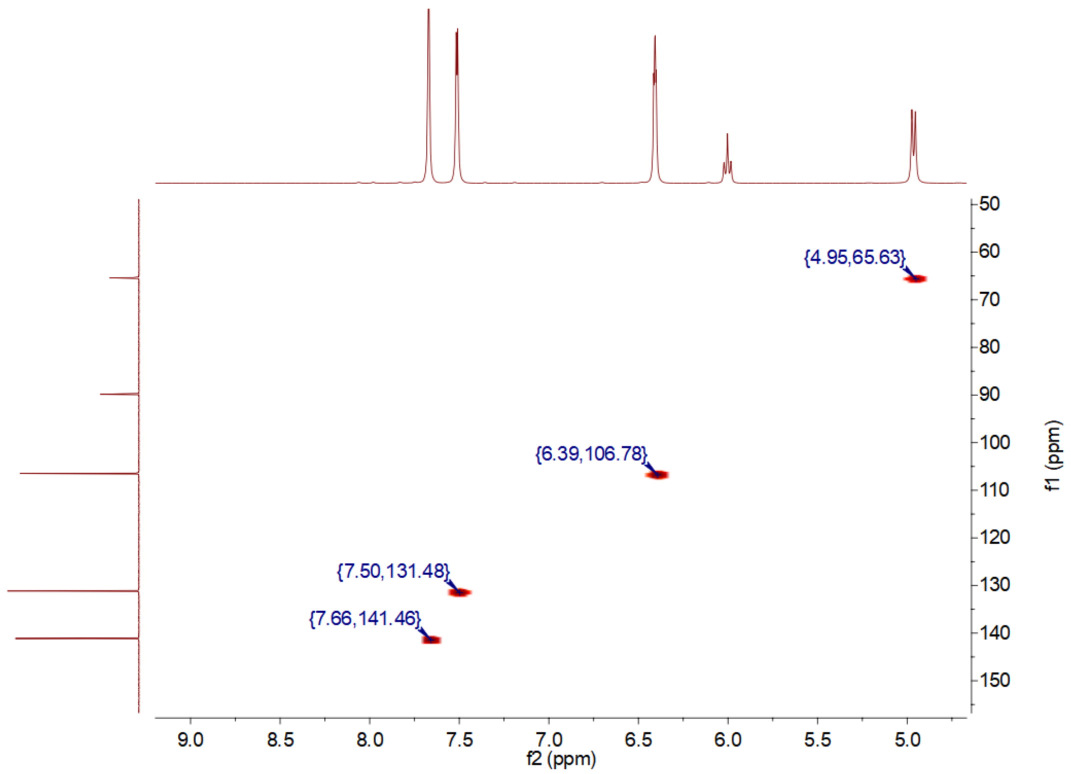

Figure S6. HSQC NMR spectrum of complex **1** in DMSO-*d*$_6$ (300 MHz).

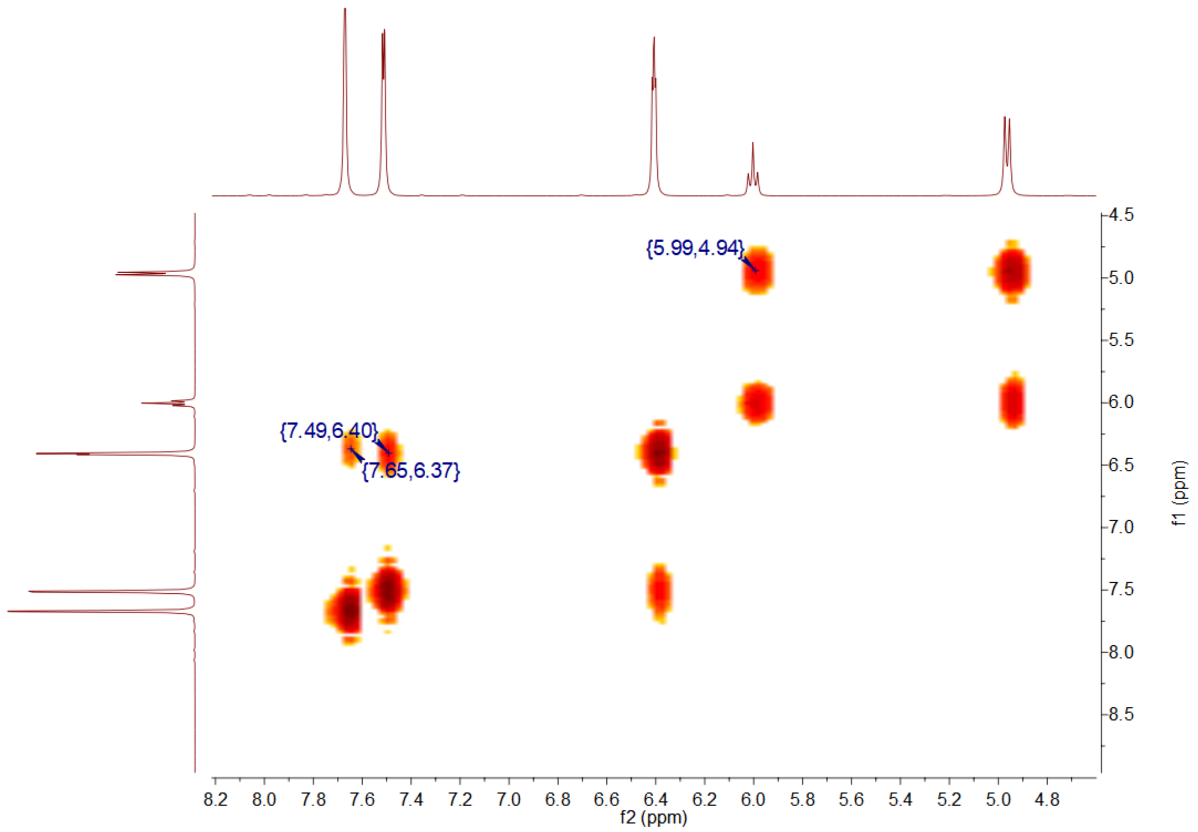

Figure S7. COSY NMR spectrum of complex **1** in DMSO-*d*$_6$ (300 MHz).

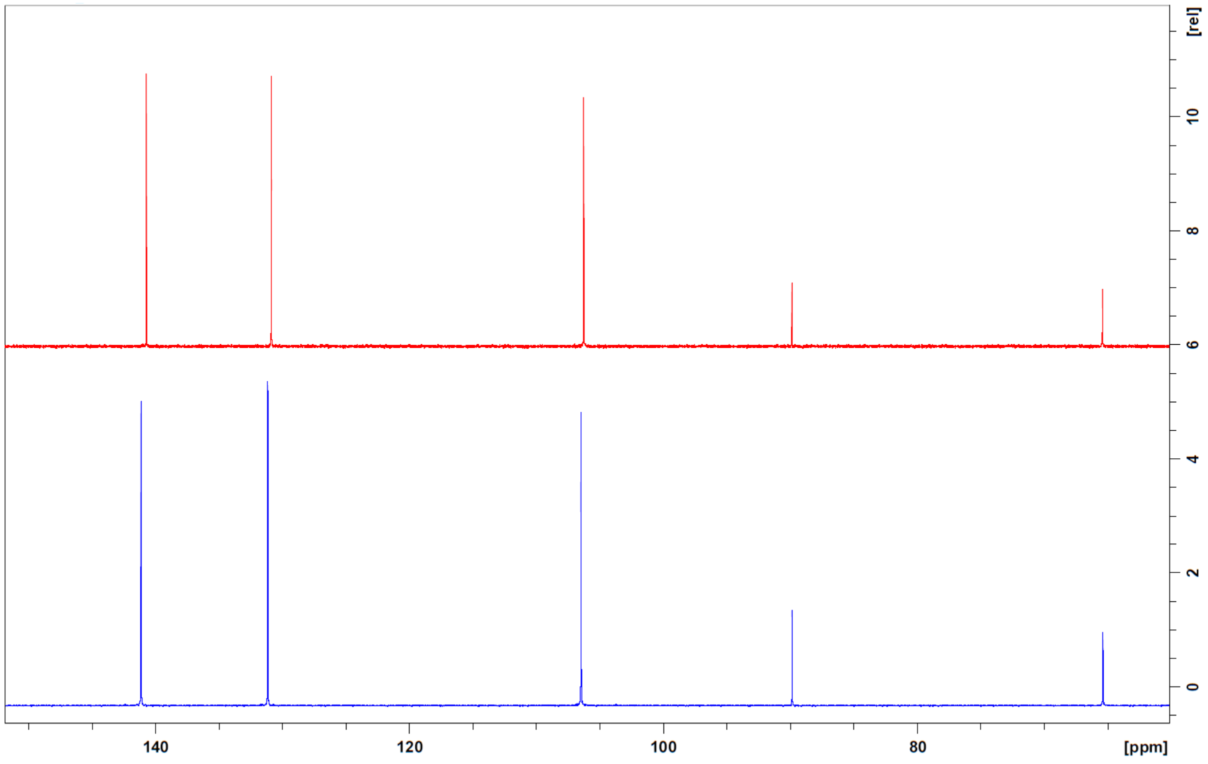

Figure S8. ^{13}C NMR spectrum of Tpm^{OH} (Top) and complex **1** (bottom) in DMSO-*d*$_6$.

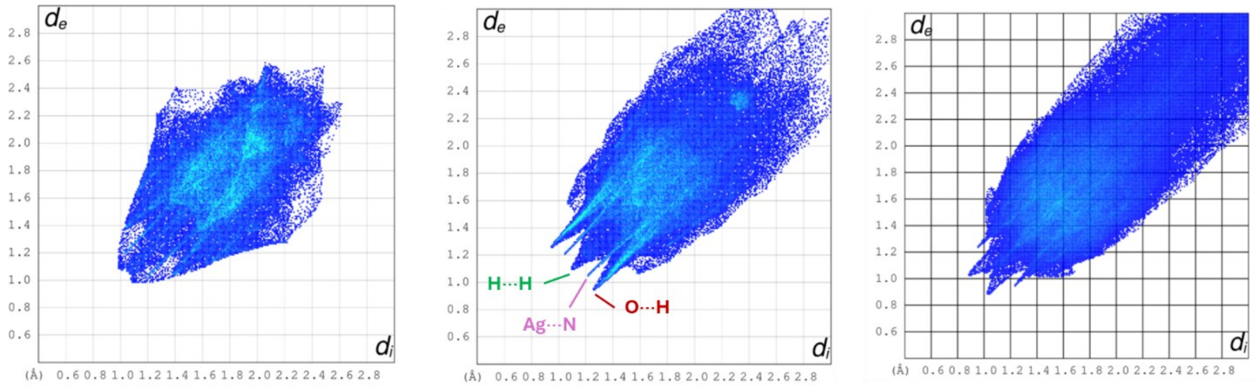

Figure S9. Overall 2D fingerprint plots for **1** (left), **2** (middle) and $[AgP_4].4TPMS.BF_4$ (right)

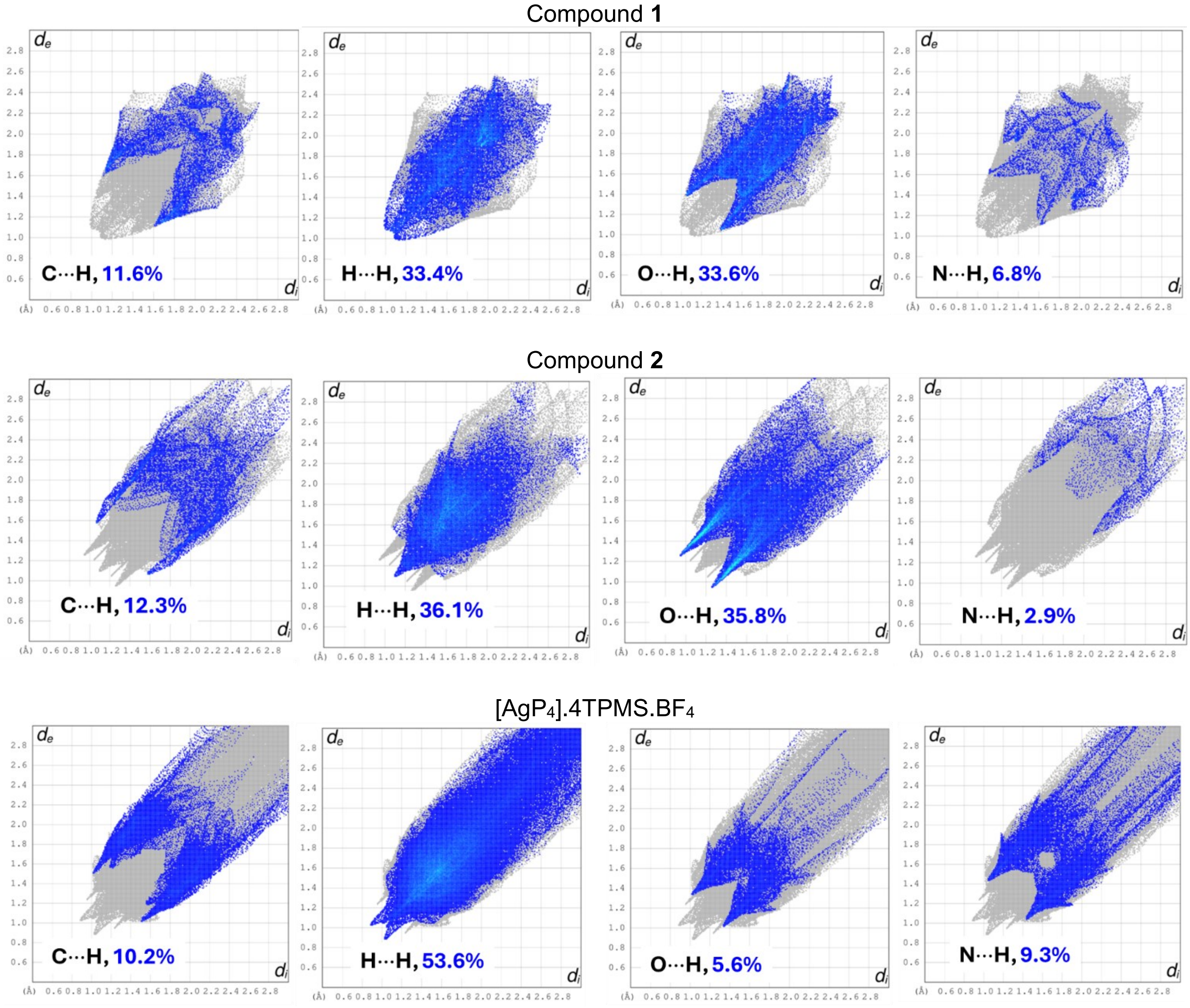

Figure S10. Specific 2D fingerprint plots for **1**, **2** and $[AgP_4].4TPMS.BF_4$
